# Successful EXCOR biventricular assist device weaning because of recovery in a child

**DOI:** 10.1177/02676591251390665

**Published:** 2025-10-16

**Authors:** Morgane Chave, Amir-Reza Hosseinpour, Fabrizio De Rita, Sylvain Mauron, Maik Meissner, Rainer Rebmann, Eleonora Dal Sasso, Stefano Di Bernardo, Maria-Helena Perez

**Affiliations:** 1 Paediatric Intensive Care Unit, Service of Paediatrics, Department of Women-Mother-Child, Lausanne University Hospital and University of Lausanne, Lausanne, Switzerland; 2 Department of Cardiac Surgery, Lausanne University Hospital and University of Lausanne, Lausanne, Switzerland; 3 Adult Congenital and Pediatric Heart Surgery Unit, Transplantation and Mechanical Assist Devices, 105565 Freeman Hospital, Newcastle-Upon-Tyne, UK; 4 Department of Anaesthesia, Lausanne University Hospital and University of Lausanne, Lausanne, Switzerland; 5 500244 Berlin Heart GmbH, Berlin, Germany; 6 Paediatric Cardiology Unit, Service of Paediatrics, Department of Women-Mother-Child, Lausanne University Hospital and University of Lausanne, Lausanne, Switzerland

**Keywords:** ventricular assist device, Berlin heart, EXCOR VAD, myocarditis, myocardial recovery, VAD weaning

## Abstract

**Introduction:**

Ventricular assist devices (VADs) are used in children usually as a bridge to transplantation and occasionally also as a bridge to recovery, e.g., for acute myocarditis. However, in cases of recovery, weaning criteria are not well defined and protocols are scarce.

**Case report:**

We report our first experience with weaning of biventricular assist device (EXCOR® Pediatric, Berlin Heart GmbH, Germany) in a 3-year-old boy due to recovery.

**Discussion:**

The weaning and explantation processes were based on clinical experience, local hospital practices, and collaboration with the Berlin Heart team.

**Conclusion:**

Regular echocardiographic assessments, weaning attempts, and downsizing of the EXCOR pumps may facilitate device weaning.

## Introduction

Ventricular assist devices (VADs) are used in children primarily as bridge to transplantation and occasionally as bridge to recovery, e.g., for acute myocarditis.^1–6^ However, in cases of recovery, the criteria for weaning off these devices are not well defined, and protocols are limited.^3,7–10^ We report our experience with weaning a biventricular assist device (BVAD, EXCOR^®^ Pediatric, Berlin Heart GmbH, Germany), in a 3-year-old boy. Written informed consent for this publication was obtained from the patient’s parents.

## Case report

A 3-year-old boy with a history of chemotherapy for lymphoma and viral illness (enterovirus on nasal swab) was admitted to the Paediatric Intensive Care Unit (PICU) with refractory cardiogenic shock requiring veno-arterial extracorporeal membrane oxygenation. Daily echocardiography revealed persistent left ventricular dysfunction. On day 11, EXCOR left ventricular assist device (LVAD) implantation was planned. However, right ventricular function deteriorated during the procedure, requiring implantation of two EXCOR pumps (25 mL): an LVAD set to a rate of 72 bpm (3 L/min/m^2^) and a right VAD (RVAD) at 60 bpm (2.3 L/min/m^2^). The patient was extubated on day 31, receiving salicylic acid, dipyridamole, and anticoagulation therapy (initially heparin, then bivalirudin). Myocardial biopsy showed myocarditis.

Over the following 6 months, myocardial function improved enough for us to consider weaning the BVAD. After discussions with Berlin Heart (BH), BVAD support was gradually reduced while monitoring progress with daily echocardiography. Both pumps’ rates were gradually decreased to 60 bpm (2.1 L/min/m^2^), the lower limit considered safe to avoid blood stagnation in the chambers.

Following BH’s recommendations, we conducted an initial BVAD weaning assessment in the PICU under sedation, without any inotropic support, after administering additional heparin (100 IU/kg). The RVAD rate was reduced to 30 bpm for 5 minutes and then to 5 bpm, with echocardiographic assessments at each stage. This process was carried out also for the LVAD. Then the pump system was manually operated once every 30 s. Cardiac function remained stable on echocardiography.

On day 209, a detailed weaning assessment was conducted in the catheterization laboratory under general anaesthesia with laryngeal mask. An infusion of phenylephrine (0.2 mcg/kg/min) was administered to maintain stable blood pressure. The rates of both pumps were reduced gradually to 5/min for 20 min. Cardiac index (CI) was 2.5 L/min/m^2^, wedge and central venous pressures were 10 and 11 mmHg respectively, and central venous oxygen saturation was 66%. These parameters were considered borderline. After further discussion with BH, we implemented a slower weaning strategy, downsizing both pumps to 15 ml on day 231.

Over the following weeks, the rates of both sides were reduced from 90 bpm (CI of 1.9 L/min/m^2^) to 60 bpm (CI of 1.3 L/min/m^2^). Additionally, levosimendan (0.2 mcg/kg/min over 24 h, every 3 weeks) was administered twice. Follow-up echocardiography indicated stability.

Another weaning assessment on day 265 lasted 40 min and showed CI of 2.9 L/min/m^2^, central venous oxygen saturation of 77%, and wedge and central venous pressures of 11 and 12 mmHg respectively.

Weaning and device explantation was done on day 288. Induction of anaesthesia (midazolam, ketamine, sufentanyl, atracurium) was followed with sevoflurane inhalation (continued on bypass via the circuit). The patient’s thorax, abdomen and the four EXCOR VAD cannulae were disinfected and prepared. Heparin (300 IU/kg) was administered. Both EXCOR VADs were stopped and disconnected from their respective cannulae, which were immediately connected to the cardiopulmonary bypass circuit: the right atrial cannula connected to the venous drainage of the circuit, and the aortic conduit (a Dacron tube anastomosed to the ascending aorta) to the arterial side of the circuit. Bypass was started. The remaining two EXCOR cannulae were used for additional drainage.

After resternotomy, standard bypass cannulae were placed in the distal ascending aorta and right atrium; the bypass circuit was disconnected from the EXCOR VAD cannulae and connected to these new cannulae. The aorta was cross-clamped and del Nido cardioplegia administered into the ascending aorta via the aortic Dacron conduit. The LVAD apex cannula was removed and its site repaired with 4/0 polypropylene sutures and Bioglue® (Artivion, Inc., Kennesaw, US). The RVAD atrial cannula was removed and its site repaired with 4/0 polypropylene sutures. The RVAD arterial cannula was removed from the pulmonary artery and its site repaired with 6/0 polypropylene sutures.

The heart was de-aired via the Dacron conduit. The cross-clamp was removed. The heart gradually adopted a normal sinus rhythm. The patient was weaned off bypass on norepinephrine 0.3mcg/kg/min, dopamine 5 mcg/kg/min and milrinone 0.5 mcg/kg/min. Cardiac function was adequate, with slight septal dyskinesia and minimal tricuspid regurgitation. The bypass cannulae were removed. The aortic Dacron tube was transected and oversewn. Bypass and cross-clamp times were 226 and 62min respectively. Protamine and fibrinogen were given for haemostasis. The sternum was closed. Sevoflurane was discontinued and a dexmedetomidine infusion started. The patient was extubated and transferred to PICU on non-invasive ventilation.

Postoperative course was unremarkable. The patient was discharged from the PICU 1 week later, receiving the same medications as before weaning: enalapril, torasemide, spironolactone, propranolol, and monthly levosimendan. He was discharged home 2 months later. Cardiac function remains stable 2years later. He receives Entresto® (Novartis Pharma, Switzerland) and monthly levosimendan prophylactically as he needs chemotherapy (with risk of cardiotoxicity) for recurrent lymphoma.

## Discussion

Weaning off VADs is complex with no established protocols. We did this based on clinical judgment and collaboration with the BH support team. Nevertheless, other protocols, also based on clinical common sense, have been reported.^3,7–10^ These protocols are similar to ours in that they are based on trials of gradually reducing and stopping the mechanical support while using both echocardiography and catheterization to assess cardiac recovery and establish the indication for weaning, which includes an adequate cardiac index and systemic venous oxygen saturation, and low filling pressures. However, our protocol has an additional component, namely that it includes substituting the two devices with smaller ones, thus refining the weaning process by allowing a slower and more gentle reduction of mechanical support.

## Conclusion

Echocardiographic evaluations, weaning assessments, levosimendan therapy, and downsizing of EXCOR pumps may enhance the likelihood of successful weaning.

## Data Availability

All data related to this work in contained within this article.*

## Appendix

### Abbreviations

BH: Berlin Heart GmbH
BVAD: Bi-ventricular assist devices
CI: Cardiac index
LVAD: Left ventricular assist device
PICU: Paediatric intensive care unit
RVAD: Right ventricular assist device
VADs: Ventricular assist devices
